# Draft Genome Sequence of Mycoparasite *Clonostachys rosea* Strain 67-1

**DOI:** 10.1128/genomeA.00546-15

**Published:** 2015-05-28

**Authors:** Zhan-Bin Sun, Man-Hong Sun, Shi-Dong Li

**Affiliations:** Key Laboratory of Integrated Pest Management in Crops, Ministry of Agriculture, Institute of Plant Protection, Chinese Academy of Agricultural Sciences, Beijing, China

## Abstract

*Clonostachys rosea* is a promising mycoparasite. In this study, we sequenced the draft genome of the highly effective strain 67-1 using the Illumina HiSeq 2500 sequencing platform. The genome is 55.4 Mb with a G+C content of 49.2% and provides a powerful resource for future studies on the molecular mechanisms underlying *Clonostachys rosea*’s antagonism on fungal pathogens.

## GENOME ANNOUNCEMENT

*Clonostachys rosea* (syn. *Gliocladium roseum*) is a promising biocontrol agent against various plant pathogenic fungi due to mycoparasitism, antibiosis, competition, and induced plant resistance (1–3), and it has been used in the management of plant fungal diseases in the greenhouse and the field (4–6). Isolate 67-1, originally obtained from soil in a vegetable yard in Hainan Province, China, using a sclerotia baiting method (7), shows great potential as a biocontrol agent against a variety of fungal plant pathogens, and its use has resulted in increased crop yields (8–10). The genome sequence of strain 67-1 will undoubtedly prove useful for future studies on the molecular mechanisms underlying its antagonism on fungal pathogens. To the best of our knowledge, this is the first reported draft genome sequence of *C. rosea* in China.

*C. rosea* 67-1 was inoculated onto potato dextrose agar (BD Difco, USA) and incubated at 26°C for 7 days. Sufficient mycelia were collected and transferred onto a sieve with 25-µm pores using a sterile spatula and washed five times with sterile distilled water to remove fungal spores. Genomic DNA was extracted using a Biospin Fungus Genomic DNA extraction kit (Bioer Technology Co., Ltd., USA) according to the manufacturer’s instructions. Three paired-end libraries of 170,300 and 500 bp and one mate-pair library of 5 kb were constructed for high-throughput sequencing on the Illumina HiSeq 2500 platform. The raw data generated were filtered using FASTX-Toolkit software, and clean data were assembled using Velvet version 1.2.03 (11).

The genome sequence coverage was at least 150×. The 5-kb mate-pair library included 8,626,470-bp clean reads, while 9,064,212-, 11,510,231-, and 8,769,057-bp clean reads were obtained from the 170-, 300-, and 500-bp paired-end libraries, respectively, for subsequent genome assembly. The draft genome of *C. rosea* 67-1 comprised 1,552 contigs, and further generated 475 scaffolds, with a longest size of 2,741,725 bp and an *N*_50_ value of 569,486 bp. In total, 20,747 genes were predicted by the Fgenesh software (12), in which 13,474 were found to be homologous with the nonredundant (NR) database.

*C. rosea* is a mycoparasite in which cell wall-degrading enzymes such as chitinases and serine proteases play an important role in parasitizing on plant fungal pathogens (13, 14). A total of 55 genes encoding cell wall-degrading enzymes were identified and annotated, including 32 glucanases, 14 proteinases, and 9 chitinases. In addition, 19 genes encoding ABC transporters were identified that might be involved in detoxifying the toxic metabolites secreted by the pathogenic fungi (15). Our future work will focus on identifying mycoparasitism-associated genes and exploring the molecular mechanisms underpinning the parasitic capabilities of *C. rosea*.

### Nucleotide sequence accession numbers. 

This whole-genome shotgun project has been deposited at DDBJ/EMBL/GenBank under the accession number JYFM00000000. The version described in this paper is JYFM02000000.
